# Estimating the benefits of obesity prevention on productivity: an Australian perspective

**DOI:** 10.1038/s41366-022-01133-z

**Published:** 2022-05-11

**Authors:** Kirthi Menon, Barbora de Courten, Zanfina Ademi, Alice J. Owen, Danny Liew, Ella Zomer

**Affiliations:** 1 School of Public Health and Preventive Medicine, Monash University, Melbourne, VIC Australia; 2 Department of Medicine, School of Clinical Sciences, Monash University, Melbourne, VIC Australia; 3 Centre for Medicine Use and Safety, Faculty of Pharmacy and Pharmaceutical Sciences, Monash University, Melbourne, VIC Australia; 4 Adelaide Medical School, University of Adelaide, Adelaide, SA Australia

**Keywords:** Preventive medicine, Epidemiology

## Abstract

**Background/objectives:**

Obesity poses one of the biggest public health challenges globally. In addition to the high costs of obesity to the healthcare system, obesity also impacts work productivity. We aimed to estimate the benefits of preventing obesity in terms of years of life, productivity-adjusted life years (PALYs) and associated costs over 10 years.

**Subjects/methods:**

Dynamic life table models were constructed to estimate years of life and PALYs saved if all new cases of obesity were prevented among Australians aged 20–69 years from 2021 to 2030. Life tables were sex specific and the population was classified into normal weight, overweight and obese. The model simulation was first undertaken assuming currently observed age-specific incidences of obesity, and then repeated assuming all new cases of obesity were reduced by 2 and 5%. The differences in outcomes (years of life, PALYs, and costs) between the two modelled outputs reflected the potential benefits that could be achieved through obesity prevention. All outcomes were discounted by 5% per annum.

**Results:**

Over the next 10 years, 132 million years of life and 81 million PALYs would be lived by Australians aged 20–69 years, contributing AU$17.0 trillion to the Australian economy in terms of GDP. A 5% reduction in new cases of obesity led to a gain of 663 years of life and 1229 PALYs, equivalent to AU$262 million in GDP.

**Conclusions:**

Prevention of obesity is projected to result in substantial economic gains due to improved health and productivity. This further emphasises the need for public health prevention strategies to reduce this growing epidemic.

## Introduction

The prevalence of overweight and obesity globally has tripled over the last 40 years, with current figures indicating that 40% of the world’s adult population is overweight and 13% obese [1]. In Australia, two thirds of the adult population are overweight or obese [2]. Obesity contributes to the development of many chronic diseases, including type 2 diabetes (T2DM), cardiovascular disease, obstructive sleep apnoea, several cancers, osteoarthritis, non-alcoholic fatty liver disease and resultant cirrhosis, kidney disease and depression. There is also ample evidence suggesting that obesity and its associated complications increase mortality and reduce life expectancy. Data from the Global Burden of Disease from 2015 [3] showed that a high BMI resulted in 7% excess mortality from any cause. In Australia, 7% of the total burden of disease is due to overweight and obesity [2].

While the health impact of obesity is well recognised, it is also important to consider the economic consequences of obesity, which include direct healthcare costs associated with medical resource utilisation, as well as indirect costs, which represent those incurred outside of the healthcare system. Reduced work productivity represents a large component of indirect costs, and is caused by absenteeism (time off work due to ill health), presenteeism (reduced productivity while at work) and reduced workforce participation [4]. Furthermore, research has shown that individuals with obesity are less likely to be employed compared to normal weight individuals [5].

Overall, total direct healthcare costs and government subsidies were higher among people with overweight and obesity compared to people with normal weight [6]. A study in Australia reported the total annual direct healthcare costs for a person of normal weight to be AU$1998 and AU$2501 for a person with obesity [6].

In Australia in 2011–2012, obesity was estimated to cost the Australian economy AU$8.6 billion, which comprised AU$3.8 billion in direct healthcare costs and AU$4.8 billion in indirect costs [2].

While studies have reported the direct and indirect costs of obesity, to date, no Australian studies have described the impact of obesity on work productivity at a population level using a dynamic model which captures movement across weight categories, including future cases of obesity. Work productivity drives a country’s economy through income earnings, tax revenue and gross domestic product (GDP) [7], making it imperative to understand and quantify the future economic impact of obesity on productivity. The latter can be quantified in terms of productivity-adjusted life years (PALYs), a novel measure which adjusts years lived to account for impairment in productivity [8–10], akin to quality-adjusted life years (QALYs) adjusting for impairment in quality of life.

In the present study, we sought to forecast the potential benefits of obesity prevention on work productivity and the economy of Australia over the 10-year period from 2021 to 2030.

## Material/subjects and methods

### Models

Dynamic life table models (Appendix 1) were constructed to estimate years of life lived, PALYs lived and associated economic costs in Australia from 2021 to 2030 for the Australian population of working age (20 to 69 years) [11]. Life tables were age and sex specific, and were stratified into three categories; people with normal weight, people with overweight and people with obesity, defined by body mass indices (BMI) of 18.5 to <25 kg/m^2^, 25 to <30 kg/m^2^ and ≥30 kg/m^2^, respectively [12].

To capture the dynamic nature of population projections, the models:

- Included population changes over the follow-up period, including projections in death rates and migration.
- Included movement in and out of weight categories, based on expected trends.
- Allowed subjects to enter the model when they reached age 20 years within the 10-year time horizon, and exit the model when they reached 70 years.

See Appendix 1 for an example of movements among the three weight categories.

To estimate the potential benefits of obesity prevention, the model simulations were repeated assuming the incidence of obesity was reduced by 2 and 5%, with redistribution of subjects into the normal and overweight categories. To note, changes in prevalence of obesity were not explored. The difference in modelled outcomes between the currently predicted situation, and situations that assumed a reduction in future cases of obesity reflected the impact of preventing obesity. All years of life lived, PALYs lived and costs incurred from the second year of the model simulation (2021) onwards were discounted by 5% per annum as per current guidelines [13]. Discounting is standard in economic modelling and down-adjusts the value of benefits and costs acquired in the future. The approach accounts for the fact that costs and other benefits are ascribed lesser value by consumers the further into the future they are acquired [14].

### Model population

The demographic profile of the model population was based on Australian population characteristics in 2020 [15]. The prevalence of underweight, normal weight, overweight and obesity was obtained from the National Health Survey 2017–18, stratified according to sex and age-group [16] (Appendix 2). These estimates were used to distribute subjects into the weight categories in the baseline year of 2020 (Appendix 3). To estimate prevalence for individual ages, polynomial functions were derived using the midpoints of each age-group (Appendix 4 and Appendix 5). People in the 2017–2018 National Health Survey who were classified as underweight (BMI < 18.5 kg/m^2^) were excluded from the analyses. This is due to the complex nature and increased mortality risk associated with being underweight [17], and the lack of data on transitions (movement) to other weight categories. Additionally, the proportion of underweight Australians in the model was small: <5%, <2% and <1% in the age groups 20–29, 30–34 and 35–69 years, respectively.

### Transition between BMI categories

Movement in and out of the normal, overweight and obese weight categories was informed by 4-year longitudinal data from the Household, Income and Labour Dynamics in Australia (HILDA) study [18]. The formulae to calculate the annual transition probabilities are included in Appendix 6. These transition probabilities were not influenced by age and sex, and therefore remained constant across all ages and sex.

When the incidence of obesity was hypothetically reduced, only movement from the normal weight and overweight categories into the obese category was reduced. Movement could still occur among the other weight categories and from obese to overweight or normal weight.

### Migration

Data on net overall migration were sourced from the Australian Bureau of Statistics (ABS), with the projected number of immigrants and emigrants stratified by sex and single year of age [19] (Appendix 7). Net migration represents the difference between immigration and emigration (specific to the year and sex). Because there were no data on the weight of immigrants and emigrants, we assumed that the proportional distribution of these people into the three weight categories was the same as for the current resident Australian population.

### Mortality risks

Age and sex-specific projected mortality data were obtained from the ABS [19] (Appendix 8). Mortality for the sub-population with obesity were estimated considering the total mortality rate and the increased risk of mortality conferred by obesity. A systematic review and meta-analysis by the Global BMI Mortality Collaboration estimated that the hazard ratio for people with obesity compared to those in the normal BMI range in Australia and New Zealand is 1.44 (95% confidence interval (CI): 1.34 to 1.54) [20]. As the hazard ratio for overweight people was minimal and statistically non-significant (HR 1.01, 95% CI: 0.93 to 1.08) [20], we assumed that the risk of mortality for those who were overweight was the same as those of normal weight. Of note, the mortality hazard ratio for people with obesity remained constant across age and sex groups. The formulae used to calculate the mortality rates for people with obesity and people who were non-obese (normal/overweight) are included in Appendix 9.

### The effect of obesity on productivity

As Australian data were lacking, information on absenteeism was obtained from a study by Andreyeva et al. which reported absenteeism by BMI groups using US National Health and Nutrition Examination Survey (NHANES) 1998–2008 data [21]. The study found that people with normal weight miss an average of 4.25 days annually, people with overweight miss 4.48 days annually and people with obesity miss 5.62 days annually [21]. Presenteeism data were not available, and hence we assumed that presenteeism did not differ according to weight category.

Productivity was measured in terms of PALYs [8–10]. PALYs are calculated by multiplying years lived by a productivity index, which varies between 0 (no productivity) and 1.0 (full productivity). One PALY is equivalent to one fully productive year. We assumed that there were 240 total working days per year (assuming 4 weeks of annual leave per year and 5 working days per week). Applying the average number of workdays missed reported by Andreyeva et al. [21] generated productivity indices of 0.982 for people with normal weight, 0.981 for people with overweight, and 0.977 for people with obesity.

PALYs were calculated by multiplying years of life lived by the productivity indices specific to each BMI category (described above). PALYs were then adjusted to account for workforce participation in different age groups and sex (Appendix 10), which took into account the average hours worked (as a proportion of full-time hours of 40 h per week) in those employed. Each PALY was ascribed a financial value in terms of GDP, equivalent to the GDP for a full-time worker. Using trend data on GDP per hour worked (Appendix 11) and assuming a full-time worker works 1920 h per year (40 h × 48 weeks), the value of the PALY ranged from AU$199,562 in 2021 to AU$220,286 in 2030. PALYs generated in each year of the model were multiplied by the GDP for a full-time worker for the specific year to calculate their economic value.

### Scenario analyses

Scenario analyses were undertaken to assess the impact of changing key data inputs on model outcomes. These comprised: varying absenteeism data using the lower and upper bounds of the CIs as reported by Andreyeva et al.; varying the HRs for mortality associated with obesity using the lower and upper bounds of the CIs; applying a constant GDP per EFT over the 10-year time horizon of AU$199,562 in line with the 2021 value; and assessing the effect of a 3% annual discount rate.

Key data inputs, their base-case values and the values used in scenario analyses are summarised in Table 1.

**Table 1:** Key data inputs, their base-case values and the values used in scenario analyses.

Parameters	Cohort/population	Base-case values	Confidence intervals	
			Lower bound	Upper bound
Productivity indices^a^	With obesity	0.977	0.972	0.981
	With overweight	0.981	0.979	0.984
	With normal weight	0.982	0.980	0.985
Hazard ratio for mortality with obesity^b^	With obesity	1.44	1.34	1.54
Annual discount rate	Total	5%	3%	–

^a^Productivity indices were derived from absenteeism data reported by Andreyeva et al. where fewer days off work means greater productivity and therefore a higher productivity index and vice versa.

^b^The hazard ratio of mortality for the population with obesity was compared to the population with normal weight (reference population). As the hazard ratio for the population with overweight was non-significant, we assumed that the risk of mortality was equal to the population with normal weight.

A scenario where there was no ‘recovery’ of obesity, hence no movement from the obese weight category to overweight and normal weight categories, was also explored. In addition, we assessed the impact of obesity prevention in this scenario.

## Results

### Years of life lived

Table 2 summarises the projected years of life lived assuming current trajectories of obesity for the Australian population aged between 20 and 69 years from 2021 to 2030. The total years of life lived over the decade was estimated to be 132,040,252 (discounted). Overall, females lived more years of life compared to males (66,493,172 versus 65,547,081). With a reduction in the incidence of obesity by 2 and 5%, the total years of life lived for the population increased by 265 and 663 life years, respectively

**Table 2:** Total years of life lived, PALYs and value of PALYs estimated for the Australian working-age population from 2021 to 2030 (values for the total population).

Year	Years of life lived	PALYs	Value of PALYs^a^
2021	15,583,957	9,636,915	$1,923,157,871,084
2022	15,011,855	9,277,567	$1,872,809,452,263
2023	14,453,452	8,927,275	$1,822,655,254,952
2024	13,913,026	8,589,112	$1,773,391,823,052
2025	13,389,766	8,263,956	$1,725,286,509,097
2026	12,882,629	7,949,860	$1,678,018,356,545
2027	12,394,126	7,646,484	$1,631,590,915,741
2028	11,922,672	7,354,505	$1,586,224,489,216
2029	11,465,604	7,073,263	$1,541,853,884,983
2030	11,023,166	6,803,637	$1,498,746,714,447
Total	132,040,252	81,522,574	$17,053,735,271,380

All outcomes reported were subject to an annual discount rate of 5%; costs are reported in Australian dollars (AU$); due to rounding of data presented in this table, totals may not precisely match.

*PALYs* productivity-adjusted life years.

^a^The value of PALYs were estimated using gross domestic product (GDP) per equivalent full-time worker (EFT).

### Productivity-adjusted life years

Tables 2 and 3 summarise the projected PALYs lived for the Australian population of working age from 2021 to 2030, and the potential PALYs gained assuming reductions in the future incidence of obesity (prevention of obesity). Assuming no change in the future incidence of obesity, there would be 81,522,574 PALYs (discounted) lived, with more PALYs lived by men than women (48,269,875 versus 33,252,698). If new cases of obesity were reduced by 2 and 5%, 491 and 1,229 PALYs would be gained, respectively. The equivalent gains in GDP amounted to AU$105 and AU$262 million, respectively.

**Table 3:** Scenario analyses to assess the impact of reducing the incidence of obesity on total years of life lived, PALYs and value of PALYs for the Australian working-age population from 2021 to 2030 (values for the total population).

Obesity incidence	Years of life lived	Years of life saved	PALYs	PALYs saved	Value of PALYs^a^	Value of PALYs saved
100% (base case)	132,040,252		81,522,574		$17,053,735,271,380	–
98%	132,040,517	265	81,523,064	491	$17,053,839,957,357	$104,685,977
95%	132,040,915	663	81,523,802	1229	$17,053,997,456,963	$262,185,583

All outcomes were subject to an annual discount rate of 5%; costs are reported in Australian dollars (AU$); due to rounding of data presented in this table, values may not precisely match.

*PALYs* productivity-adjusted life years.

^a^The value of PALYs were estimated using gross domestic product (GDP) per equivalent full-time worker (EFT).

Discounted results stratified by sex are provided in Appendix 12.

### Scenario analyses

Table 4 summarises the results of the scenario analyses. The modelled results were most sensitive to productivity indices, temporal growth in GDP and the annual discount rate. At the lower and upper bounds of the 95% CI estimates for absenteeism, there was a potential loss or gain of AU$52 billion compared to the base case. If we assumed no temporal growth in GDP, there would be a loss of approximately AU$800 billion. Lastly, a reduction in the annual discount rate to 3% led to a gain of AU$1.8 trillion.

**Table 4:** One-way sensitivity and scenario analyses to assess the impact of the uncertainties surrounding key input parameters on years of life lived, PALYs and the value of PALYs for the Australian working-age population from 2021 to 2030 (values for the total population).

		Years of life lived	Years of life gained/lost	PALYs lived	PALYs gained/lost	Value of PALYs^a^	Value of PALYs gained/lost
Base case^b^		132,040,252		81,522,574		$17,053,735,271,380	
One-way sensitivity analysis							
Productivity indices^c^							
1a	Lower bound	132,040,252	N/A	81,275,885	−246,688	$17,002,147,350,544	−$51,587,920,835
1b	Upper bound	132,040,252	N/A	81,769,478	+246,904	$17,105,368,304,068	+$51,633,032,689
Hazard ratio for mortality with obesity							
2a	Lower bound	132,075,264	+35,012	81,539,936	+17,362	$17,057,424,035,182	+$3,688,763,802
2b	Upper bound	132,005,303	−34,949	81,505,236	−17,337	$17,050,051,866,285	−$3,683,405,094
Scenario analysis							
3	No temporal growth in GDP	132,040,252	N/A	81,522,574	N/a	$16,268,772,448,145	−$784,962,823,234
4	Annual discount rate of 3%	146,102,529	+14,062,277	90,201,276	+8,678,703	$18,902,035,239,631	+$1,848,299,968,251
5	Recovery of obesity omitted^d^	132,021,912	−18,340	81,493,289	−29,285	$17,047,484,354,010	−$6,250,917,369

All outcomes were subject to an annual discount rate of 5%; all costs are reported in Australian dollars (AU$); due to rounding of data presented in this table, values may not precisely match.

+ Gained, − lost, *N/A* not applicable, *PALYs* productivity-adjusted life years.

^a^The value of PALYs were estimated using gross domestic product (GDP) per equivalent full-time worker (EFT).

^b^The base case represents the results for the total population assuming the current trajectory of obesity over the 10-year period of 2021 to 2030.

^c^Upper and lower bounds of the productivity indices were estimated using the lower and upper bounds of the 95% confidence intervals for absenteeism data reported by Andreyeva et al. where fewer days off work means greater productivity and the inverse for more days off work.

^d^People with obesity could not ‘recover’, and therefore there was no movement from the obese weight category to overweight or normal weight categories.

When ‘recovery’ of obesity was omitted, there was a loss of ~AU$6 billion, compared to the base case. A 5% reduction in the incidence of obesity resulted in gains of AU$271 million.

## Discussion

Findings from our study highlight the potential gains that could be achieved through prevention of obesity in terms of years of life, productivity and the economy over the next 10 years, demonstrating the importance of obesity prevention. The results will also help inform policies and public health preventive programmes. In light of this, strategies that address the problem of obesity (both prevention and treatment) should be considered as a long-term investment rather than an expenditure. That is, spending on population prevention or intervention strategies to reduce the number of people with overweight and obesity represents an upfront cost that in the long term is likely to save money from the reduced downstream healthcare costs and increased work productivity.

Initiatives for the prevention of obesity have been undertaken at many levels (national and global) in view of the rising incidence of obesity. Lifestyle interventions (dietary counselling and physical exercise with or without a behavioural modification component) have been shown to significantly reduce body weight and cardiovascular risk factors in people with overweight and obesity at an average follow-up of 3 years [22]. A study in people of South Asian descent reported a mean weight loss of 1.13 kg in the group randomised to a lifestyle intervention compared to a mean weight gain of 0.51 kg in the control group at 3 years [23]. A meta-analysis of randomised controlled trials focused on lifestyle interventions targeting weight loss observed a reduction in all-cause mortality in adults with obesity [24]. However, existing evidence regarding weight-loss maintenance has been mixed. There is evidence to suggest that people who lose weight using lifestyle interventions regain 30–35% of their lost weight in the year after treatment [25], while a study by Funk et al. reported that ~40% of participants had gained back some weight after a community-based weight-loss intervention [26]. Nevertheless, studies have also demonstrated long-term weight-loss maintenance following an intervention [27–29]. This was evident in a meta-analysis of studies in the US demonstrating weight-loss maintenance 5 years after completion of a structured weight-loss programme [27]. Medical and surgical options for the treatment of obesity provide an alternative option to individuals who fail to achieve weight loss through lifestyle interventions, but may not be cost-effective [30, 31].

There are no directly comparable studies to ours, but our findings are in general accord with those of other studies that have explored the impact of obesity on productivity and the economy. Obesity was associated with over 4 million days per year lost from the workplace among working Australians [32]. A recently published systematic review of productivity loss due to overweight and obesity by Goettler et al. [33] reported evidence of significant indirect costs associated with overweight and obesity. In contrast, people with overweight or obesity who achieved 5% or greater weight-loss benefited from a reduction in absenteeism by 0.26 days per month and lower presenteeism levels compared to those who did not achieve similar weight loss [34].

Recognising the urgency of the situation, workplace wellness programmes [35] and nutrition-related workplace initiatives have been undertaken to help increase work productivity [36], but effective management strategies to address this problem are still needed [33]. A significant proportion of school-age children with obesity are obese as adults [37], and therefore obesity prevention needs to start in early childhood. Healthy food choices, nutritional education to parents and children, access and opportunity to engage in physical activity and screen time reduction are some of the efforts required for the early prevention of childhood obesity [38]. On a larger scale, the sugar reduction programme in the UK which aims to reduce sugar content in the food industry by 20% and legislative change like the sugar tax in the UK or Mexico, are all efforts aimed at reducing the overall sugar consumption of the population including in children which in the long-term is expected to improve health outcomes. A modelling study of people from England aged 4–80 years predicted that if the sugar reduction programme were implemented successfully, there would be a reduction in obesity by 5.5% in people with obesity aged 4–10 and 19–80 years and by 2.2% in children with obesity aged 11–18 years [39]. Additionally, there would be a net healthcare saving of £285.8 million [39]. The restriction of marketing unhealthy food to children through media (such as restricted times for advertisements) are among efforts to reduce childhood obesity. This has been made mandatory in several countries, but not Australia. Interestingly, while there are many studies of interventions to address obesity, there are limited studies on obesity prevention, particularly in adults, or studies that correlate obesity prevention with obesity incidence. Lemmens et al. identified nine studies in a systematic review on the efficacy of interventions for obesity prevention in adults [40]. Three of these reported a significant change in BMI. Nevertheless, there is a lack of evidence in the literature regarding the type of interventions needed to see a reduction in the incidence of obesity.

### Strengths and limitations

To our knowledge, this study is the first to describe the benefits of obesity prevention on productivity in the Australian working-age population using a dynamic modelling approach. The use of PALYs provides an objective measure to estimate the economic impact of obesity at a population level. The dynamic nature of the model allowed for a more realistic projection of future trends in obesity by incorporating population changes over time and movement in and out of weight categories. These measures added to the robustness of the model. Additionally, the transition between weight categories is from an Australian study which is representative of the Australian population.

Several limitations of our study warrant mention. First, due to the nature of our model structure and best available evidence, the prevalence of obesity (calculated as the number of people with obesity in the total population) reduced over time from 31.05% in 2020 to 24.66% in 2030. This is contrary to projected trends in obesity prevalence, with obesity prevalence expected to increase to 35% in 2025 [41]. This is likely due to the (1) the model structure which follows people in each weight category separately, (2) the incidence of obesity and ‘recovery’ from obesity to overweight and normal weight categories is derived from self-reported data which is likely to underestimate incidence and overestimate ‘recovery’, and (3) mortality rates applied to people with obesity are higher than contemporary studies which show that mortality for people with obesity is improving and people with obesity are living longer. Bhaskaran et al. undertook a population-based cohort study using UK primary care data from 3,632,674 people and found that for every 5 kg/m^2^ increase in BMI above 25 kg/m^2^, there was a 21% increase in all-cause mortality [42]. However, data from the Global BMI Mortality Collaboration (which we employed in our model), found that for every 5 kg/m^2^ increase in BMI above 25 kg/m^2^, there was a 31% increase in Australia/NZ and 39% increase in Europe (which included data from the UK) [20]. We explored our model assumptions, and the prevalence only increased when the ‘recovery’ of obesity was omitted, with the prevalence of obesity increasing from 31.05% in 2020 to 33.73% in 2030. The increased prevalence of obesity resulted in fewer years of life and PALYs than the base-case as people with obesity were at a higher risk of death and had greater productivity impacts. When we reduced the incidence of obesity by 5% in this scenario, the years of life and PALYs saved were 673 and 1271, respectively, with estimated savings of AU$271 million in GDP. These savings are slightly more than what was predicted in our base-case model and therefore, our model underestimates the impact of obesity prevention over the next 10 years if obesity prevalence continues to rise. Second, the HILDA study used self-reported measures of weight and height to estimate BMI. It is well known that people tend to overestimate their height and underestimate their weight, which means that we likely under- estimated the incidence, and therefore burden of obesity. Furthermore, transition probabilities among weight categories and mortality hazard ratios by BMI were constant across age and sex due to the lack of age and sex-specific data. Additionally, we disregarded movement from the underweight to other weight categories (as these data were not available), but such transitions would be minimal, and therefore would not change the overall findings from this study. Third, due to a lack of recent studies on the productivity impact of obesity specific to the Australian population, we based our estimates on US sources [21]. Additionally, the reported productivity impacts were not age or sex specific, nor did they include information related to job-type. Employment data relating to part time and full-time employment or employment levels (ie: workforce participation) were not specific to BMI categories and only available for the general population. With studies already demonstrating less workforce participation among individuals with obesity [43], these assumptions would have led to an under-estimation of the impact obesity has on work productivity, PALYs and subsequently GDP. Furthermore, the true impact of obesity on productivity was not fully captured since presenteeism data were unavailable and therefore not included in our estimation of productivity indices. Fourth, differences in workforce participation (or workforce dropout) and productivity indices for the different age categories, as well as unpaid work (which are often predominantly undertaken by females and therefore likely to influence female workforce participation) and maternity leave were not accounted for in our models as relevant input data were not available. Next, we assumed that migrant populations were distributed into weight categories in the same proportions as the resident Australian population in 2020, which may be inaccurate. The ‘healthy immigrant effect’ theory assumes immigrant heath is better than that of comparable native born people [44]. One study has found that US-born Asian-Americans are significantly more likely to be overweight or obese than those foreign born [45]. Additionally, the risk of being overweight or obese was directly related to the length of time spent in the US. This assumption may have inflated the observed effect of obesity on work productivity since Australia’s immigrants in 2019 were highest from regions in Asia [46] which have a lower prevalence of obesity [47]. We also recognise that because of the current coronavirus disease 2019 (COVID-19) pandemic, migration rates have been affected. However, since these numbers are small, we anticipate that these assumptions would only have affected our findings minimally. Last, our analyses were limited to incident obesity and therefore only assessed the impact of preventing new cases of obesity. We acknowledge that some public health strategies that target obesity are also likely to reduce the incidence of overweight (movement from normal to overweight categories) which we did not account for in the analyses. Furthermore, this model captures the economic impact in terms of productivity and did not consider healthcare costs. None of the above limitations however would have changed our conclusion that measures to counter obesity are likely to be economically justifiable.

## Conclusions

The impact of obesity is not limited to health consequences and medical expenditure. Our study highlights the potential gains in productivity and the Australian economy that could be achieved via prevention of obesity over the next 10 years. In addition to direct costs, the productivity loss associated with obesity makes a stronger case for evidence-based public health initiatives like the implementation of the sugar reduction programme in the UK which is likely to be cost saving in the long run.

## Supplementary information

Supplemental Material

## Data Availability

All relevant data were sourced from publicly available sources and are described/included within the paper and its Supplementary Data.
