# Supplementary material for: Estimating the benefits of obesity prevention on productivity: an Australian perspective
Source: Int J Obes (Lond). 2022 May 11;46(8):1463–9. doi: 10.1038/s41366-022-01133-z (PMC9092329; doi:10.1038/s41366-022-01133-z)
Supplement: Supplementary file 1 — Supplemental Material [file 41366_2022_1133_MOESM1_ESM.docx]

**Supplementary Appendices**

Estimating the benefits of obesity prevention on productivity: An Australian perspective.

Kirthi Menon1 Barbora de Courten2, Zanfina Ademi1,3, Alice J Owen1, Danny Liew1,4, Ella Zomer1

1. School of Public Health and Preventive Medicine, Monash University, Melbourne, Australia
2. Department of Medicine, School of Clinical Sciences, Monash University, Melbourne, Australia
3. Centre for Medicine Use and Safety, Faculty of Pharmacy and Pharmaceutical Sciences, Monash University, Melbourne, Australia
4. Adelaide Medical School, University of Adelaide, Adelaide, Australia

**Supplementary Appendices**

Appendix 1: Movement between health states in the dynamic model.

Appendix 2: Prevalence (%) of the Australian population with underweight, normal weight, overweight and obesity by age-group for a) males and b) females (results from the National Health Survey 2018).

Appendix 3: Prevalence (%) of underweight, normal weight, overweight and obesity for the Australian population by single year of age and sex (derived using data from Appendix 4 and 5).

Appendix 4: Scatter plots of the age-group specific prevalence of underweight, normal weight, overweight and obesity for the Australian population for a) males and b) females (derived from data in Appendix 2).

Appendix 5: Polynomial functions used to determine the probability of i) underweight, ii) overweight, iii) obese and iv) normal weight for a) males and b) females. The probability of being normal weight was estimated as the reciprocal of the other weight categories.

Appendix 6: Annual probability of transitioning in and out of weight categories.

Appendix 7: Data on net overall migration (projections from the Australian Bureau of Statistics) by age for a) males and b) females

Appendix 8: Mortality rates (%) for the total population by age (projections from the Australian Bureau of Statistics) for a) males and b) females.

Appendix 9: Formulae used to estimate mortality rates for people with and without obesity.

Appendix 10: Estimated equivalent full-time workers using the Australian Bureau of Statistics labour force statistics for a) males and b) females.

Appendix 11: GDP per hour worked (projections derived from the Australian Bureau of Statistics trend data from 1975 to 2018).

Appendix 12: The discounted years of life lived, PALYs and value of PALYs for the Australian working age from 2021 to 2030 for males and females assuming a) current trajectory of incident obesity, b) a 2% reduction in incident obesity and c) a 5% reduction in incident obesity.

Appendix 1: Movement between health states in the dynamic model.


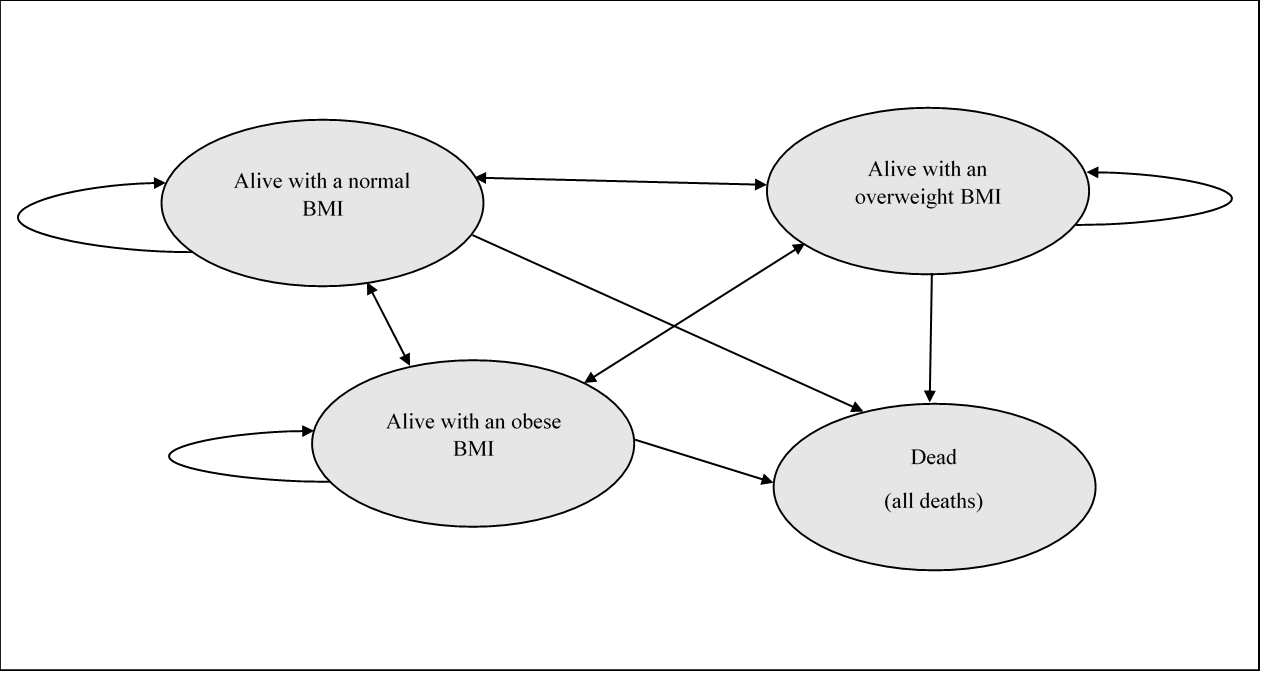


To estimate the total number of people in a particular health state the approach below was used:

Eg: To estimate the total number of 22-year-old females with normal weight in 2022:

[the number of 21 year old females with normal weight in 2021] + [the number of 22 year old female migrants with normal weight in 2022] + [the number of 22 year old females moving into the normal weight category (movement in from other weight categories) in 2022] – [the number of 22 year old females who are no longer normal weight (movement out into other weight categories) in 2022] – [the number of 21 year old females with normal weight who died in 2021].

Appendix 2: Prevalence (%) of the Australian population with underweight, normal weight, overweight and obesity by age-group for a) males and b) females (results from the National Health Survey 2018).

1. Males

| **Age group (years)** | **Underweight** | **Normal weight** | **Overweight** | **Obese** |
| --- | --- | --- | --- | --- |
| 18-24 | 3.6% | 44.5% | 35.0% | 18.1% |
| 25-34 | 0.6% | 32.9% | 42.2% | 24.6% |
| 35-44 | 0.3% | 22.5% | 45.4% | 32.0% |
| 45-54 | 0.3% | 16.9% | 43.0% | 40.6% |
| 55-64 | 0.7% | 15.7% | 42.2% | 41.6% |
| 65-74 | 0.2% | 16.3% | 40.9% | 42.2% |
| 75 and over | 0.9% | 24.0% | 44.9% | 30.0% |

1. Females

| **Age group (years)** | **Underweight** | **Normal weight** | **Overweight** | **Obese** |
| --- | --- | --- | --- | --- |
| 18-24 | 5.0% | 56.1% | 26.0% | 13.5% |
| 25-34 | 2.4% | 48.6% | 26.0% | 22.9% |
| 35-44 | 0.5% | 38.9% | 30.7% | 29.6% |
| 45-54 | 1.5% | 33.4% | 30.7% | 34.5% |
| 55-64 | 0.9% | 32.7% | 28.4% | 38.1% |
| 65-74 | 0.3% | 26.5% | 34.4% | 38.7% |
| 75 and over | 2.1% | 28.2% | 34.1% | 35.4% |

Appendix 3: Prevalence (%) of underweight, normal weight, overweight and obesity for the Australian population by single year of age and sex (derived using data from Appendix 4 and 5).

| **Age (years)** | **Male** | | | | **Females** | | | | |
| --- | --- | --- | --- | --- | --- | --- | --- | --- | --- |
|  | **Under-weight** | **Normal weight** | **Over-weight** | **Obese** | **Under-weight** | **Normal weight** | **Over-weight** | **Obese** |  |
| 0 | 10.48% | 63.10% | 15.08% | 11.34% | 8.16% | 61.66% | 16.01% | 14.18% |  |
| 1 | 10.21% | 64.43% | 14.87% | 10.48% | 8.28% | 63.14% | 15.83% | 12.75% |  |
| 2 | 9.94% | 65.49% | 14.81% | 9.77% | 8.35% | 64.41% | 15.72% | 11.52% |  |
| 3 | 9.66% | 66.27% | 14.88% | 9.19% | 8.40% | 65.48% | 15.66% | 10.46% |  |
| 4 | 9.37% | 66.81% | 15.07% | 8.75% | 8.41% | 66.35% | 15.67% | 9.57% |  |
| 5 | 9.07% | 67.12% | 15.39% | 8.42% | 8.39% | 67.04% | 15.73% | 8.84% |  |
| 6 | 8.77% | 67.21% | 15.81% | 8.22% | 8.34% | 67.56% | 15.85% | 8.26% |  |
| 7 | 8.46% | 67.09% | 16.33% | 8.12% | 8.26% | 67.91% | 16.00% | 7.82% |  |
| 8 | 8.15% | 66.79% | 16.94% | 8.13% | 8.16% | 68.12% | 16.21% | 7.52% |  |
| 9 | 7.83% | 66.30% | 17.63% | 8.24% | 8.04% | 68.17% | 16.45% | 7.35% |  |
| 10 | 7.51% | 65.65% | 18.39% | 8.44% | 7.89% | 68.09% | 16.73% | 7.29% |  |
| 11 | 7.20% | 64.85% | 19.22% | 8.73% | 7.72% | 67.89% | 17.04% | 7.35% |  |
| 12 | 6.88% | 63.91% | 20.11% | 9.10% | 7.54% | 67.57% | 17.38% | 7.51% |  |
| 13 | 6.56% | 62.84% | 21.05% | 9.55% | 7.34% | 67.14% | 17.75% | 7.77% |  |
| 14 | 6.24% | 61.66% | 22.03% | 10.07% | 7.13% | 66.60% | 18.15% | 8.12% |  |
| 15 | 5.93% | 60.37% | 23.05% | 10.65% | 6.90% | 65.98% | 18.56% | 8.55% |  |
| 16 | 5.61% | 58.99% | 24.10% | 11.30% | 6.67% | 65.27% | 19.00% | 9.06% |  |
| 17 | 5.30% | 57.53% | 25.17% | 12.00% | 6.42% | 64.48% | 19.45% | 9.64% |  |
| 18 | 5.00% | 55.99% | 26.26% | 12.75% | 6.17% | 63.63% | 19.92% | 10.28% |  |
| 19 | 4.70% | 54.39% | 27.37% | 13.54% | 5.91% | 62.71% | 20.40% | 10.99% |  |
| 20 | 4.40% | 52.74% | 28.47% | 14.38% | 5.64% | 61.74% | 20.88% | 11.74% |  |
| 21 | 4.11% | 51.05% | 29.58% | 15.25% | 5.37% | 60.71% | 21.37% | 12.54% |  |
| 22 | 3.83% | 49.33% | 30.69% | 16.16% | 5.10% | 59.65% | 21.87% | 13.38% |  |
| 23 | 3.56% | 47.57% | 31.78% | 17.09% | 4.83% | 58.55% | 22.37% | 14.25% |  |
| 24 | 3.29% | 45.81% | 32.86% | 18.05% | 4.56% | 57.42% | 22.87% | 15.16% |  |
| 25 | 3.03% | 44.03% | 33.92% | 19.02% | 4.28% | 56.26% | 23.37% | 16.09% |  |
| 26 | 2.77% | 42.25% | 34.96% | 20.01% | 4.01% | 55.08% | 23.86% | 17.04% |  |
| 27 | 2.53% | 40.48% | 35.98% | 21.02% | 3.75% | 53.90% | 24.35% | 18.00% |  |
| 28 | 2.29% | 38.72% | 36.96% | 22.03% | 3.48% | 52.70% | 24.84% | 18.98% |  |
| 29 | 2.07% | 36.98% | 37.90% | 23.04% | 3.23% | 51.50% | 25.31% | 19.96% |  |
| 30 | 1.85% | 35.27% | 38.82% | 24.06% | 2.97% | 50.30% | 25.78% | 20.95% |  |
| 31 | 1.65% | 33.59% | 39.69% | 25.08% | 2.73% | 49.11% | 26.23% | 21.93% |  |
| 32 | 1.45% | 31.95% | 40.51% | 26.08% | 2.49% | 47.93% | 26.67% | 22.91% |  |
| 33 | 1.27% | 30.35% | 41.30% | 27.09% | 2.26% | 46.76% | 27.10% | 23.88% |  |
| 34 | 1.09% | 28.80% | 42.03% | 28.07% | 2.04% | 45.61% | 27.51% | 24.84% |  |
| 35 | 0.93% | 27.31% | 42.72% | 29.05% | 1.83% | 44.48% | 27.91% | 25.78% |  |
| 36 | 0.78% | 25.87% | 43.35% | 30.01% | 1.63% | 43.37% | 28.29% | 26.70% |  |
| 37 | 0.63% | 24.49% | 43.93% | 30.95% | 1.44% | 42.30% | 28.66% | 27.61% |  |
| 38 | 0.50% | 23.18% | 44.46% | 31.86% | 1.26% | 41.25% | 29.00% | 28.49% |  |
| 39 | 0.38% | 21.93% | 44.93% | 32.75% | 1.09% | 40.24% | 29.33% | 29.34% |  |
| 40 | 0.28% | 20.76% | 45.35% | 33.62% | 0.93% | 39.26% | 29.64% | 30.16% |  |
| 41 | 0.18% | 19.66% | 45.71% | 34.45% | 0.79% | 38.33% | 29.93% | 30.95% |  |
| 42 | 0.09% | 18.64% | 46.01% | 35.26% | 0.66% | 37.43% | 30.20% | 31.71% |  |
| 43 | 0.02% | 17.70% | 46.26% | 36.03% | 0.54% | 36.58% | 30.45% | 32.44% |  |
| 44 | 0.00% | 16.79% | 46.45% | 36.76% | 0.43% | 35.77% | 30.68% | 33.13% |  |
| 45 | 0.00% | 15.95% | 46.58% | 37.47% | 0.34% | 35.00% | 30.88% | 33.78% |  |
| 46 | 0.00% | 15.21% | 46.66% | 38.13% | 0.26% | 34.28% | 31.07% | 34.39% |  |
| 47 | 0.00% | 14.56% | 46.69% | 38.75% | 0.19% | 33.61% | 31.24% | 34.96% |  |
| 48 | 0.00% | 14.00% | 46.66% | 39.34% | 0.13% | 32.98% | 31.39% | 35.50% |  |
| 49 | 0.00% | 13.54% | 46.58% | 39.88% | 0.09% | 32.41% | 31.52% | 35.99% |  |
| 50 | 0.00% | 13.17% | 46.45% | 40.38% | 0.06% | 31.88% | 31.62% | 36.44% |  |
| 51 | 0.00% | 12.89% | 46.28% | 40.83% | 0.04% | 31.40% | 31.71% | 36.84% |  |
| 52 | 0.00% | 12.70% | 46.05% | 41.24% | 0.04% | 30.97% | 31.79% | 37.21% |  |
| 53 | 0.00% | 12.60% | 45.79% | 41.61% | 0.05% | 30.58% | 31.84% | 37.53% |  |
| 54 | 0.00% | 12.59% | 45.48% | 41.93% | 0.06% | 30.25% | 31.88% | 37.81% |  |
| 55 | 0.00% | 12.66% | 45.14% | 42.20% | 0.09% | 29.96% | 31.90% | 38.05% |  |
| 56 | 0.00% | 12.81% | 44.76% | 42.43% | 0.14% | 29.71% | 31.90% | 38.25% |  |
| 57 | 0.01% | 13.03% | 44.35% | 42.61% | 0.19% | 29.51% | 31.89% | 38.41% |  |
| 58 | 0.07% | 13.27% | 43.91% | 42.75% | 0.25% | 29.35% | 31.87% | 38.52% |  |
| 59 | 0.13% | 13.58% | 43.45% | 42.84% | 0.32% | 29.24% | 31.84% | 38.61% |  |
| 60 | 0.20% | 13.94% | 42.97% | 42.88% | 0.39% | 29.16% | 31.80% | 38.65% |  |
| 61 | 0.28% | 14.37% | 42.48% | 42.88% | 0.48% | 29.11% | 31.75% | 38.66% |  |
| 62 | 0.36% | 14.84% | 41.97% | 42.83% | 0.57% | 29.11% | 31.69% | 38.63% |  |
| 63 | 0.44% | 15.37% | 41.46% | 42.73% | 0.67% | 29.13% | 31.62% | 38.58% |  |
| 64 | 0.53% | 15.93% | 40.95% | 42.59% | 0.78% | 29.18% | 31.55% | 38.49% |  |
| 65 | 0.61% | 16.54% | 40.44% | 42.41% | 0.89% | 29.25% | 31.48% | 38.38% |  |
| 66 | 0.70% | 17.17% | 39.94% | 42.18% | 1.00% | 29.35% | 31.41% | 38.24% |  |
| 67 | 0.79% | 17.83% | 39.46% | 41.92% | 1.11% | 29.47% | 31.34% | 38.08% |  |
| 68 | 0.88% | 18.51% | 39.01% | 41.61% | 1.23% | 29.60% | 31.27% | 37.90% |  |
| 69 | 0.96% | 19.20% | 38.58% | 41.26% | 1.35% | 29.74% | 31.21% | 37.70% |  |

Data for people aged 0 to 19 years is included as this was used in the dynamic model to estimate population projections over time.

Appendix 4: Scatter plots of the age-group specific prevalence of underweight, normal weight, overweight and obesity for the Australian population for a) males and b) females (derived from data in Appendix 2).

1. Males
2. Females

Appendix 5: Polynomial functions used to determine the probability of i) underweight, ii) overweight, iii) obese and iv) normal weight for a) males and b) females. The probability of being normal weight was estimated as the reciprocal of the other weight categories.

1. Males
   1. Underweight

y = -0.0000000124x4 + 0.0000019285x3 - 0.0000567028x2 - 0.0025738890x + 0.1047524569,

R² = 0.925

- 1. Overweight

y = 0.0000001021x4 - 0.0000169864x3 + 0.0007767865x2 - 0.0028613331x + 0.1508149196,

R² = 0.915

- 1. Obese

y = 0.0000000503x4 - 0.0000114891x3 + 0.0007510053x2 - 0.0093074855x + 0.1133882539,

R² = 0.986

- 1. Normal weight

y = 1 – (underweight + overweight + obese)

1. Females
   1. Underweight

y = -0.0000000211x4 + 0.0000038580x3 - 0.0001998275x2 + 0.0013644109x + 0.0815866799,

R² = 0.761

- 1. Overweight

y = 0.0000000384x4 - 0.0000067925x3 + 0.0003485906x2 - 0.0021248365x + 0.1600674063,

R² = 0.868

- 1. Obese

y = 0.0000000757x4 - 0.0000155000x3 + 0.0009788182x2 - 0.0152018069x + 0.1417858493,

R² = 0.991

- 1. Normal weight

y = 1 – (underweight + overweight + obese)

where y = prevalence, x = year of age

Appendix 6: Annual probability of transitioning in and out of weight categories.

| **Annual probability*** | | | |
| --- | --- | --- | --- |
| **BMI Category** | **Normal** | **Overweight** | **Obese** |
| Normal | 0.957 | 0.040 | 0.002 |
| Overweight | 0.036 | 0.934 | 0.030 |
| Obese | 0.004 | 0.039 | 0.957 |

For example, of all normal weight individuals, 4.0% will become overweight, 0.2% will become obese, and 95.7% will remain as normal weight per year.

*The annual probability from the 4-year probability reported by *Avsar et al* was calculated using the following formulae:

p = 1 - exp {-rt}

r = - [ln (1 - p)]/t

where p is the probability, r is the rate, and t is the time period of interest.

The probability of transitioning to other weight categories were not age and sex-specific, and therefore these were applied as a constant across all ages and sexes.

Appendix 7: Data on net overall migration (projections from the Australian Bureau of Statistics) by age for a) males and b) females.

1. Males

| **Age (years)** | **2021** | **2022** | **2023** | **2024** | **2025** | **2026** | **2027*** |
| --- | --- | --- | --- | --- | --- | --- | --- |
| 0 | 256 | 252 | 245 | 239 | 232 | 226 | 220 |
| 1 | 1490 | 1468 | 1428 | 1394 | 1354 | 1320 | 1280 |
| 2 | 1863 | 1835 | 1785 | 1743 | 1693 | 1650 | 1601 |
| 3 | 1957 | 1927 | 1875 | 1830 | 1778 | 1733 | 1681 |
| 4 | 1945 | 1915 | 1863 | 1819 | 1767 | 1723 | 1671 |
| 5 | 1815 | 1787 | 1739 | 1698 | 1649 | 1608 | 1559 |
| 6 | 1542 | 1518 | 1477 | 1442 | 1401 | 1366 | 1324 |
| 7 | 1345 | 1325 | 1289 | 1258 | 1222 | 1191 | 1156 |
| 8 | 1293 | 1273 | 1239 | 1209 | 1175 | 1145 | 1111 |
| 9 | 1294 | 1274 | 1240 | 1210 | 1176 | 1146 | 1112 |
| 10 | 1221 | 1203 | 1170 | 1142 | 1110 | 1082 | 1049 |
| 11 | 1208 | 1190 | 1158 | 1130 | 1098 | 1070 | 1038 |
| 12 | 1217 | 1199 | 1166 | 1138 | 1106 | 1078 | 1046 |
| 13 | 1189 | 1171 | 1139 | 1112 | 1081 | 1053 | 1022 |
| 14 | 1496 | 1473 | 1433 | 1399 | 1359 | 1325 | 1285 |
| 15 | 2114 | 2081 | 2025 | 1977 | 1921 | 1872 | 1816 |
| 16 | 2338 | 2303 | 2240 | 2187 | 2125 | 2071 | 2009 |
| 17 | 3640 | 3585 | 3488 | 3404 | 3307 | 3224 | 3127 |
| 18 | 7125 | 7017 | 6827 | 6664 | 6474 | 6312 | 6122 |
| 19 | 8090 | 7967 | 7751 | 7566 | 7351 | 7166 | 6950 |
| 20 | 6272 | 6176 | 6009 | 5866 | 5699 | 5555 | 5388 |
| 21 | 5838 | 5749 | 5593 | 5460 | 5304 | 5171 | 5015 |
| 22 | 8235 | 8110 | 7890 | 7702 | 7483 | 7295 | 7075 |
| 23 | 8501 | 8372 | 8145 | 7951 | 7725 | 7530 | 7304 |
| 24 | 5909 | 5819 | 5662 | 5527 | 5369 | 5235 | 5077 |
| 25 | 4387 | 4320 | 4203 | 4103 | 3986 | 3886 | 3769 |
| 26 | 3867 | 3809 | 3706 | 3617 | 3514 | 3426 | 3323 |
| 27 | 3434 | 3382 | 3290 | 3212 | 3120 | 3042 | 2950 |
| 28 | 3187 | 3139 | 3054 | 2981 | 2896 | 2823 | 2738 |
| 29 | 2996 | 2950 | 2870 | 2802 | 2722 | 2653 | 2574 |
| 30 | 2743 | 2702 | 2629 | 2566 | 2493 | 2430 | 2357 |
| 31 | 2249 | 2215 | 2155 | 2104 | 2044 | 1992 | 1933 |
| 32 | 2243 | 2209 | 2149 | 2098 | 2038 | 1987 | 1927 |
| 33 | 2409 | 2372 | 2308 | 2253 | 2189 | 2134 | 2070 |
| 34 | 2094 | 2062 | 2007 | 1959 | 1903 | 1855 | 1799 |
| 35 | 1784 | 1757 | 1710 | 1669 | 1621 | 1581 | 1533 |
| 36 | 1663 | 1637 | 1593 | 1555 | 1511 | 1473 | 1429 |
| 37 | 1460 | 1437 | 1398 | 1365 | 1326 | 1293 | 1254 |
| 38 | 1369 | 1348 | 1312 | 1280 | 1244 | 1213 | 1176 |
| 39 | 1268 | 1249 | 1215 | 1186 | 1152 | 1123 | 1089 |
| 40 | 1131 | 1114 | 1084 | 1058 | 1028 | 1002 | 972 |
| 41 | 960 | 945 | 920 | 898 | 872 | 850 | 825 |
| 42 | 808 | 796 | 774 | 756 | 734 | 716 | 694 |
| 43 | 791 | 778 | 757 | 739 | 718 | 700 | 679 |
| 44 | 694 | 684 | 665 | 649 | 631 | 615 | 596 |
| 45 | 579 | 570 | 555 | 541 | 526 | 513 | 497 |
| 46 | 512 | 505 | 491 | 479 | 466 | 454 | 440 |
| 47 | 386 | 380 | 370 | 361 | 351 | 342 | 332 |
| 48 | 284 | 280 | 272 | 265 | 258 | 251 | 244 |
| 49 | 238 | 234 | 228 | 222 | 216 | 211 | 204 |
| 50 | 196 | 193 | 187 | 183 | 178 | 173 | 168 |
| 51 | 164 | 161 | 157 | 153 | 149 | 145 | 141 |
| 52 | 184 | 181 | 176 | 172 | 167 | 163 | 158 |
| 53 | 141 | 139 | 135 | 132 | 128 | 125 | 121 |
| 54 | 143 | 141 | 137 | 133 | 130 | 126 | 123 |
| 55 | 53 | 52 | 51 | 49 | 48 | 47 | 45 |
| 56 | 76 | 75 | 73 | 71 | 69 | 67 | 65 |
| 57 | 111 | 109 | 106 | 103 | 100 | 98 | 95 |
| 58 | 160 | 157 | 153 | 149 | 145 | 141 | 137 |
| 59 | 188 | 185 | 180 | 176 | 171 | 167 | 162 |
| 60 | 236 | 232 | 226 | 221 | 214 | 209 | 203 |
| 61 | 267 | 263 | 256 | 249 | 242 | 236 | 229 |
| 62 | 231 | 228 | 222 | 216 | 210 | 205 | 199 |
| 63 | 265 | 261 | 254 | 248 | 241 | 235 | 228 |
| 64 | 238 | 234 | 228 | 223 | 216 | 211 | 205 |
| 65 | 112 | 110 | 107 | 105 | 102 | 99 | 96 |
| 66 | 119 | 118 | 114 | 112 | 108 | 106 | 103 |
| 67 | 128 | 126 | 123 | 120 | 116 | 114 | 110 |
| 68 | 110 | 108 | 105 | 103 | 100 | 97 | 94 |
| 69 | 78 | 77 | 75 | 73 | 71 | 69 | 67 |

1. Females

| **Age (years)** | **2021** | **2022** | **2023** | **2024** | **2025** | **2026** | **2027*** |
| --- | --- | --- | --- | --- | --- | --- | --- |
| 0 | 249 | 245 | 239 | 233 | 226 | 221 | 214 |
| 1 | 1426 | 1404 | 1366 | 1334 | 1296 | 1263 | 1225 |
| 2 | 1806 | 1778 | 1730 | 1689 | 1641 | 1600 | 1552 |
| 3 | 1908 | 1879 | 1829 | 1785 | 1734 | 1691 | 1640 |
| 4 | 1911 | 1882 | 1831 | 1787 | 1736 | 1693 | 1642 |
| 5 | 1793 | 1766 | 1718 | 1677 | 1629 | 1588 | 1540 |
| 6 | 1527 | 1504 | 1463 | 1428 | 1388 | 1353 | 1312 |
| 7 | 1348 | 1327 | 1291 | 1260 | 1224 | 1194 | 1158 |
| 8 | 1290 | 1271 | 1236 | 1207 | 1172 | 1143 | 1109 |
| 9 | 1246 | 1227 | 1194 | 1165 | 1132 | 1104 | 1070 |
| 10 | 1182 | 1164 | 1132 | 1105 | 1074 | 1047 | 1015 |
| 11 | 1126 | 1109 | 1079 | 1053 | 1023 | 998 | 968 |
| 12 | 1139 | 1121 | 1091 | 1065 | 1035 | 1009 | 978 |
| 13 | 1165 | 1147 | 1116 | 1090 | 1059 | 1032 | 1001 |
| 14 | 1456 | 1434 | 1395 | 1362 | 1323 | 1290 | 1251 |
| 15 | 2021 | 1991 | 1937 | 1890 | 1837 | 1790 | 1737 |
| 16 | 2342 | 2307 | 2244 | 2191 | 2128 | 2075 | 2012 |
| 17 | 3639 | 3584 | 3487 | 3404 | 3307 | 3224 | 3127 |
| 18 | 6694 | 6593 | 6414 | 6261 | 6083 | 5930 | 5751 |
| 19 | 7649 | 7533 | 7329 | 7154 | 6950 | 6775 | 6572 |
| 20 | 6164 | 6070 | 5906 | 5765 | 5601 | 5460 | 5295 |
| 21 | 5847 | 5758 | 5602 | 5468 | 5312 | 5179 | 5023 |
| 22 | 8396 | 8268 | 8044 | 7853 | 7629 | 7437 | 7213 |
| 23 | 8645 | 8513 | 8283 | 8085 | 7855 | 7658 | 7427 |
| 24 | 6140 | 6047 | 5883 | 5743 | 5579 | 5439 | 5275 |
| 25 | 5209 | 5130 | 4991 | 4872 | 4733 | 4614 | 4476 |
| 26 | 4879 | 4805 | 4675 | 4564 | 4433 | 4322 | 4192 |
| 27 | 4610 | 4540 | 4417 | 4312 | 4189 | 4084 | 3961 |
| 28 | 4140 | 4077 | 3967 | 3872 | 3762 | 3667 | 3557 |
| 29 | 3799 | 3741 | 3640 | 3553 | 3452 | 3365 | 3264 |
| 30 | 3415 | 3363 | 3272 | 3194 | 3103 | 3025 | 2934 |
| 31 | 2799 | 2757 | 2682 | 2618 | 2544 | 2480 | 2405 |
| 32 | 2627 | 2587 | 2517 | 2457 | 2387 | 2327 | 2257 |
| 33 | 2627 | 2587 | 2517 | 2457 | 2387 | 2327 | 2257 |
| 34 | 2380 | 2344 | 2281 | 2226 | 2163 | 2108 | 2045 |
| 35 | 2040 | 2009 | 1954 | 1908 | 1853 | 1807 | 1752 |
| 36 | 1855 | 1827 | 1778 | 1735 | 1686 | 1643 | 1594 |
| 37 | 1663 | 1638 | 1593 | 1555 | 1511 | 1473 | 1429 |
| 38 | 1499 | 1476 | 1437 | 1402 | 1362 | 1328 | 1288 |
| 39 | 1410 | 1388 | 1351 | 1319 | 1281 | 1249 | 1211 |
| 40 | 1296 | 1276 | 1242 | 1212 | 1177 | 1148 | 1113 |
| 41 | 1118 | 1101 | 1071 | 1046 | 1016 | 990 | 960 |
| 42 | 1011 | 996 | 969 | 946 | 919 | 896 | 869 |
| 43 | 926 | 912 | 887 | 866 | 841 | 820 | 795 |
| 44 | 845 | 832 | 810 | 791 | 768 | 749 | 726 |
| 45 | 679 | 669 | 650 | 635 | 617 | 601 | 583 |
| 46 | 624 | 614 | 597 | 583 | 567 | 552 | 536 |
| 47 | 485 | 478 | 465 | 454 | 441 | 430 | 417 |
| 48 | 388 | 382 | 372 | 363 | 353 | 344 | 334 |
| 49 | 352 | 347 | 337 | 329 | 320 | 312 | 303 |
| 50 | 275 | 270 | 263 | 257 | 250 | 243 | 236 |
| 51 | 253 | 249 | 242 | 236 | 230 | 224 | 217 |
| 52 | 284 | 280 | 272 | 266 | 258 | 252 | 244 |
| 53 | 265 | 261 | 254 | 248 | 241 | 235 | 228 |
| 54 | 281 | 277 | 269 | 263 | 255 | 249 | 241 |
| 55 | 228 | 224 | 218 | 213 | 207 | 202 | 196 |
| 56 | 225 | 221 | 215 | 210 | 204 | 199 | 193 |
| 57 | 289 | 284 | 277 | 270 | 262 | 256 | 248 |
| 58 | 309 | 304 | 296 | 289 | 280 | 273 | 265 |
| 59 | 343 | 337 | 328 | 320 | 311 | 303 | 294 |
| 60 | 342 | 337 | 328 | 320 | 311 | 303 | 294 |
| 61 | 362 | 356 | 347 | 339 | 329 | 321 | 311 |
| 62 | 313 | 308 | 299 | 292 | 284 | 277 | 269 |
| 63 | 297 | 292 | 284 | 277 | 269 | 263 | 255 |
| 64 | 257 | 253 | 246 | 241 | 234 | 228 | 221 |
| 65 | 183 | 181 | 176 | 172 | 167 | 162 | 158 |
| 66 | 178 | 176 | 171 | 167 | 162 | 158 | 153 |
| 67 | 157 | 155 | 150 | 147 | 143 | 139 | 135 |
| 68 | 140 | 138 | 134 | 131 | 127 | 124 | 120 |
| 69 | 115 | 113 | 110 | 107 | 104 | 101 | 98 |

A positive number indicated net influx and a negative number net efflux. Net efflux was only seen in older people (aged >80 years) and therefore, only positive numbers (net influx) are included in our model due to the age profile of our model cohort. Data for people aged 0 to 19 years is included as this was used in the dynamic model to estimate population projections over time.

*Beyond 2027, the net overall migration for 2027 was applied.

Appendix 8: Mortality rates (%) for the total population by age (projections from the Australian Bureau of Statistics) for a) males and b) females.

1. Males

| **Age (years)** | **2020** | **2021** | **2022** | **2023** | **2024** | **2025** | **2026** | **2027** | **2028** | **2029** |
| --- | --- | --- | --- | --- | --- | --- | --- | --- | --- | --- |
| 0 | 0.31% | 0.30% | 0.29% | 0.29% | 0.28% | 0.27% | 0.27% | 0.26% | 0.26% | 0.25% |
| 1 | 0.03% | 0.03% | 0.03% | 0.02% | 0.02% | 0.02% | 0.02% | 0.02% | 0.02% | 0.02% |
| 2 | 0.02% | 0.01% | 0.01% | 0.01% | 0.01% | 0.01% | 0.01% | 0.01% | 0.01% | 0.01% |
| 3 | 0.01% | 0.01% | 0.01% | 0.01% | 0.01% | 0.01% | 0.01% | 0.01% | 0.01% | 0.01% |
| 4 | 0.01% | 0.01% | 0.01% | 0.01% | 0.01% | 0.01% | 0.01% | 0.01% | 0.01% | 0.01% |
| 5 | 0.01% | 0.01% | 0.01% | 0.01% | 0.01% | 0.01% | 0.01% | 0.01% | 0.01% | 0.01% |
| 6 | 0.01% | 0.01% | 0.01% | 0.01% | 0.01% | 0.01% | 0.01% | 0.01% | 0.01% | 0.01% |
| 7 | 0.01% | 0.01% | 0.01% | 0.01% | 0.01% | 0.01% | 0.01% | 0.01% | 0.01% | 0.01% |
| 8 | 0.01% | 0.01% | 0.01% | 0.01% | 0.01% | 0.01% | 0.01% | 0.01% | 0.01% | 0.01% |
| 9 | 0.01% | 0.01% | 0.01% | 0.01% | 0.01% | 0.01% | 0.01% | 0.01% | 0.01% | 0.01% |
| 10 | 0.01% | 0.01% | 0.01% | 0.01% | 0.01% | 0.01% | 0.01% | 0.01% | 0.01% | 0.01% |
| 11 | 0.01% | 0.01% | 0.01% | 0.01% | 0.01% | 0.01% | 0.01% | 0.01% | 0.01% | 0.01% |
| 12 | 0.01% | 0.01% | 0.01% | 0.01% | 0.01% | 0.01% | 0.01% | 0.01% | 0.01% | 0.01% |
| 13 | 0.01% | 0.01% | 0.01% | 0.01% | 0.01% | 0.01% | 0.01% | 0.01% | 0.01% | 0.01% |
| 14 | 0.02% | 0.02% | 0.02% | 0.02% | 0.01% | 0.01% | 0.01% | 0.01% | 0.01% | 0.01% |
| 15 | 0.02% | 0.02% | 0.02% | 0.02% | 0.02% | 0.02% | 0.02% | 0.02% | 0.02% | 0.02% |
| 16 | 0.03% | 0.03% | 0.03% | 0.03% | 0.03% | 0.03% | 0.03% | 0.03% | 0.03% | 0.03% |
| 17 | 0.04% | 0.04% | 0.03% | 0.03% | 0.03% | 0.03% | 0.03% | 0.03% | 0.03% | 0.03% |
| 18 | 0.04% | 0.04% | 0.04% | 0.04% | 0.04% | 0.04% | 0.04% | 0.04% | 0.04% | 0.04% |
| 19 | 0.05% | 0.05% | 0.05% | 0.05% | 0.05% | 0.05% | 0.04% | 0.04% | 0.04% | 0.04% |
| 20 | 0.05% | 0.05% | 0.05% | 0.05% | 0.05% | 0.05% | 0.05% | 0.05% | 0.05% | 0.05% |
| 21 | 0.06% | 0.05% | 0.05% | 0.05% | 0.05% | 0.05% | 0.05% | 0.05% | 0.05% | 0.05% |
| 22 | 0.06% | 0.06% | 0.05% | 0.05% | 0.05% | 0.05% | 0.05% | 0.05% | 0.05% | 0.05% |
| 23 | 0.06% | 0.06% | 0.06% | 0.06% | 0.05% | 0.05% | 0.05% | 0.05% | 0.05% | 0.05% |
| 24 | 0.06% | 0.06% | 0.06% | 0.06% | 0.06% | 0.05% | 0.05% | 0.05% | 0.05% | 0.05% |
| 25 | 0.06% | 0.06% | 0.06% | 0.06% | 0.06% | 0.06% | 0.05% | 0.05% | 0.05% | 0.05% |
| 26 | 0.06% | 0.06% | 0.06% | 0.06% | 0.06% | 0.06% | 0.06% | 0.06% | 0.05% | 0.05% |
| 27 | 0.06% | 0.06% | 0.06% | 0.06% | 0.06% | 0.06% | 0.06% | 0.06% | 0.06% | 0.06% |
| 28 | 0.07% | 0.06% | 0.06% | 0.06% | 0.06% | 0.06% | 0.06% | 0.06% | 0.06% | 0.06% |
| 29 | 0.07% | 0.07% | 0.07% | 0.07% | 0.06% | 0.06% | 0.06% | 0.06% | 0.06% | 0.06% |
| 30 | 0.07% | 0.07% | 0.07% | 0.07% | 0.07% | 0.07% | 0.07% | 0.07% | 0.07% | 0.07% |
| 31 | 0.08% | 0.08% | 0.08% | 0.08% | 0.07% | 0.07% | 0.07% | 0.07% | 0.07% | 0.07% |
| 32 | 0.08% | 0.08% | 0.08% | 0.08% | 0.08% | 0.08% | 0.08% | 0.08% | 0.08% | 0.08% |
| 33 | 0.09% | 0.09% | 0.09% | 0.09% | 0.09% | 0.09% | 0.08% | 0.08% | 0.08% | 0.08% |
| 34 | 0.10% | 0.09% | 0.09% | 0.09% | 0.09% | 0.09% | 0.09% | 0.09% | 0.09% | 0.09% |
| 35 | 0.10% | 0.10% | 0.10% | 0.10% | 0.10% | 0.10% | 0.10% | 0.10% | 0.10% | 0.10% |
| 36 | 0.11% | 0.11% | 0.11% | 0.11% | 0.11% | 0.10% | 0.10% | 0.10% | 0.10% | 0.10% |
| 37 | 0.11% | 0.11% | 0.11% | 0.11% | 0.11% | 0.11% | 0.11% | 0.11% | 0.11% | 0.11% |
| 38 | 0.12% | 0.12% | 0.12% | 0.12% | 0.12% | 0.12% | 0.12% | 0.12% | 0.12% | 0.12% |
| 39 | 0.13% | 0.13% | 0.13% | 0.13% | 0.13% | 0.13% | 0.13% | 0.13% | 0.13% | 0.13% |
| 40 | 0.14% | 0.14% | 0.14% | 0.14% | 0.14% | 0.14% | 0.14% | 0.14% | 0.14% | 0.14% |
| 41 | 0.15% | 0.15% | 0.15% | 0.15% | 0.15% | 0.15% | 0.15% | 0.15% | 0.15% | 0.15% |
| 42 | 0.16% | 0.16% | 0.16% | 0.16% | 0.16% | 0.16% | 0.16% | 0.16% | 0.16% | 0.16% |
| 43 | 0.17% | 0.17% | 0.17% | 0.17% | 0.17% | 0.17% | 0.17% | 0.17% | 0.17% | 0.17% |
| 44 | 0.19% | 0.18% | 0.18% | 0.18% | 0.18% | 0.18% | 0.18% | 0.18% | 0.18% | 0.18% |
| 45 | 0.20% | 0.20% | 0.20% | 0.20% | 0.20% | 0.19% | 0.19% | 0.19% | 0.19% | 0.19% |
| 46 | 0.21% | 0.21% | 0.21% | 0.21% | 0.21% | 0.21% | 0.21% | 0.21% | 0.21% | 0.21% |
| 47 | 0.23% | 0.23% | 0.23% | 0.23% | 0.23% | 0.23% | 0.23% | 0.23% | 0.23% | 0.23% |
| 48 | 0.25% | 0.25% | 0.25% | 0.25% | 0.25% | 0.25% | 0.25% | 0.25% | 0.25% | 0.25% |
| 49 | 0.27% | 0.27% | 0.27% | 0.27% | 0.27% | 0.27% | 0.27% | 0.27% | 0.27% | 0.27% |
| 50 | 0.29% | 0.29% | 0.29% | 0.29% | 0.29% | 0.29% | 0.29% | 0.29% | 0.29% | 0.29% |
| 51 | 0.32% | 0.32% | 0.32% | 0.32% | 0.31% | 0.31% | 0.31% | 0.31% | 0.31% | 0.31% |
| 52 | 0.34% | 0.34% | 0.34% | 0.34% | 0.34% | 0.34% | 0.34% | 0.34% | 0.34% | 0.34% |
| 53 | 0.37% | 0.37% | 0.37% | 0.37% | 0.37% | 0.37% | 0.37% | 0.36% | 0.36% | 0.36% |
| 54 | 0.41% | 0.40% | 0.40% | 0.40% | 0.40% | 0.40% | 0.40% | 0.40% | 0.40% | 0.40% |
| 55 | 0.44% | 0.44% | 0.44% | 0.44% | 0.43% | 0.43% | 0.43% | 0.43% | 0.43% | 0.43% |
| 56 | 0.48% | 0.47% | 0.47% | 0.47% | 0.47% | 0.47% | 0.47% | 0.47% | 0.47% | 0.46% |
| 57 | 0.52% | 0.51% | 0.51% | 0.51% | 0.51% | 0.51% | 0.51% | 0.50% | 0.50% | 0.50% |
| 58 | 0.56% | 0.56% | 0.55% | 0.55% | 0.55% | 0.55% | 0.55% | 0.54% | 0.54% | 0.54% |
| 59 | 0.61% | 0.60% | 0.60% | 0.59% | 0.59% | 0.59% | 0.59% | 0.59% | 0.58% | 0.58% |
| 60 | 0.65% | 0.65% | 0.64% | 0.64% | 0.64% | 0.63% | 0.63% | 0.63% | 0.63% | 0.62% |
| 61 | 0.71% | 0.70% | 0.69% | 0.69% | 0.68% | 0.68% | 0.68% | 0.67% | 0.67% | 0.67% |
| 62 | 0.76% | 0.75% | 0.74% | 0.74% | 0.73% | 0.73% | 0.72% | 0.72% | 0.72% | 0.71% |
| 63 | 0.82% | 0.81% | 0.80% | 0.80% | 0.79% | 0.78% | 0.78% | 0.77% | 0.77% | 0.77% |
| 64 | 0.89% | 0.87% | 0.87% | 0.86% | 0.85% | 0.84% | 0.84% | 0.83% | 0.83% | 0.82% |
| 65 | 0.96% | 0.94% | 0.94% | 0.93% | 0.92% | 0.91% | 0.90% | 0.90% | 0.89% | 0.89% |
| 66 | 1.04% | 1.02% | 1.01% | 1.00% | 0.99% | 0.99% | 0.98% | 0.97% | 0.96% | 0.96% |
| 67 | 1.14% | 1.12% | 1.11% | 1.09% | 1.08% | 1.07% | 1.06% | 1.06% | 1.05% | 1.04% |
| 68 | 1.25% | 1.22% | 1.21% | 1.20% | 1.19% | 1.17% | 1.16% | 1.15% | 1.15% | 1.14% |
| 69 | 1.37% | 1.35% | 1.33% | 1.32% | 1.30% | 1.29% | 1.27% | 1.26% | 1.26% | 1.25% |

1. Females

| **Age (years)** | **2020** | **2021** | **2022** | **2023** | **2024** | **2025** | **2026** | **2027** | **2028** | **2029** |
| --- | --- | --- | --- | --- | --- | --- | --- | --- | --- | --- |
| 0 | 0.30% | 0.29% | 0.28% | 0.28% | 0.27% | 0.27% | 0.26% | 0.26% | 0.25% | 0.25% |
| 1 | 0.02% | 0.02% | 0.02% | 0.02% | 0.02% | 0.02% | 0.02% | 0.02% | 0.02% | 0.02% |
| 2 | 0.01% | 0.01% | 0.01% | 0.01% | 0.01% | 0.01% | 0.01% | 0.01% | 0.01% | 0.01% |
| 3 | 0.01% | 0.01% | 0.01% | 0.01% | 0.01% | 0.01% | 0.01% | 0.01% | 0.01% | 0.01% |
| 4 | 0.01% | 0.01% | 0.01% | 0.01% | 0.01% | 0.01% | 0.01% | 0.01% | 0.01% | 0.01% |
| 5 | 0.01% | 0.01% | 0.01% | 0.01% | 0.01% | 0.01% | 0.01% | 0.01% | 0.01% | 0.01% |
| 6 | 0.01% | 0.01% | 0.01% | 0.01% | 0.01% | 0.01% | 0.01% | 0.01% | 0.01% | 0.01% |
| 7 | 0.01% | 0.01% | 0.01% | 0.01% | 0.01% | 0.01% | 0.01% | 0.01% | 0.01% | 0.01% |
| 8 | 0.01% | 0.01% | 0.01% | 0.01% | 0.01% | 0.01% | 0.01% | 0.01% | 0.01% | 0.01% |
| 9 | 0.01% | 0.01% | 0.01% | 0.01% | 0.01% | 0.01% | 0.01% | 0.01% | 0.01% | 0.01% |
| 10 | 0.01% | 0.01% | 0.01% | 0.01% | 0.01% | 0.01% | 0.01% | 0.01% | 0.01% | 0.01% |
| 11 | 0.01% | 0.01% | 0.01% | 0.01% | 0.01% | 0.01% | 0.01% | 0.01% | 0.01% | 0.01% |
| 12 | 0.01% | 0.01% | 0.01% | 0.01% | 0.01% | 0.01% | 0.01% | 0.01% | 0.01% | 0.01% |
| 13 | 0.01% | 0.01% | 0.01% | 0.01% | 0.01% | 0.01% | 0.01% | 0.01% | 0.01% | 0.01% |
| 14 | 0.01% | 0.01% | 0.01% | 0.01% | 0.01% | 0.01% | 0.01% | 0.01% | 0.01% | 0.01% |
| 15 | 0.02% | 0.01% | 0.01% | 0.01% | 0.01% | 0.01% | 0.01% | 0.01% | 0.01% | 0.01% |
| 16 | 0.02% | 0.02% | 0.02% | 0.02% | 0.02% | 0.02% | 0.02% | 0.02% | 0.02% | 0.02% |
| 17 | 0.02% | 0.02% | 0.02% | 0.02% | 0.02% | 0.02% | 0.02% | 0.02% | 0.02% | 0.02% |
| 18 | 0.02% | 0.02% | 0.02% | 0.02% | 0.02% | 0.02% | 0.02% | 0.02% | 0.02% | 0.02% |
| 19 | 0.02% | 0.02% | 0.02% | 0.02% | 0.02% | 0.02% | 0.02% | 0.02% | 0.02% | 0.02% |
| 20 | 0.02% | 0.02% | 0.02% | 0.02% | 0.02% | 0.02% | 0.02% | 0.02% | 0.02% | 0.02% |
| 21 | 0.02% | 0.02% | 0.02% | 0.02% | 0.02% | 0.02% | 0.02% | 0.02% | 0.02% | 0.02% |
| 22 | 0.02% | 0.02% | 0.02% | 0.02% | 0.02% | 0.02% | 0.02% | 0.02% | 0.02% | 0.02% |
| 23 | 0.02% | 0.02% | 0.02% | 0.02% | 0.02% | 0.02% | 0.02% | 0.02% | 0.02% | 0.02% |
| 24 | 0.03% | 0.02% | 0.02% | 0.02% | 0.02% | 0.02% | 0.02% | 0.02% | 0.02% | 0.02% |
| 25 | 0.03% | 0.03% | 0.03% | 0.03% | 0.03% | 0.03% | 0.02% | 0.02% | 0.02% | 0.02% |
| 26 | 0.03% | 0.03% | 0.03% | 0.03% | 0.03% | 0.03% | 0.03% | 0.03% | 0.03% | 0.03% |
| 27 | 0.03% | 0.03% | 0.03% | 0.03% | 0.03% | 0.03% | 0.03% | 0.03% | 0.03% | 0.03% |
| 28 | 0.03% | 0.03% | 0.03% | 0.03% | 0.03% | 0.03% | 0.03% | 0.03% | 0.03% | 0.03% |
| 29 | 0.03% | 0.03% | 0.03% | 0.03% | 0.03% | 0.03% | 0.03% | 0.03% | 0.03% | 0.03% |
| 30 | 0.04% | 0.04% | 0.03% | 0.03% | 0.03% | 0.03% | 0.03% | 0.03% | 0.03% | 0.03% |
| 31 | 0.04% | 0.04% | 0.04% | 0.04% | 0.04% | 0.04% | 0.04% | 0.04% | 0.04% | 0.04% |
| 32 | 0.04% | 0.04% | 0.04% | 0.04% | 0.04% | 0.04% | 0.04% | 0.04% | 0.04% | 0.04% |
| 33 | 0.05% | 0.05% | 0.05% | 0.05% | 0.04% | 0.04% | 0.04% | 0.04% | 0.04% | 0.04% |
| 34 | 0.05% | 0.05% | 0.05% | 0.05% | 0.05% | 0.05% | 0.05% | 0.05% | 0.05% | 0.05% |
| 35 | 0.05% | 0.05% | 0.05% | 0.05% | 0.05% | 0.05% | 0.05% | 0.05% | 0.05% | 0.05% |
| 36 | 0.06% | 0.06% | 0.06% | 0.06% | 0.06% | 0.06% | 0.06% | 0.06% | 0.06% | 0.06% |
| 37 | 0.06% | 0.06% | 0.06% | 0.06% | 0.06% | 0.06% | 0.06% | 0.06% | 0.06% | 0.06% |
| 38 | 0.07% | 0.07% | 0.07% | 0.07% | 0.07% | 0.07% | 0.07% | 0.07% | 0.07% | 0.07% |
| 39 | 0.07% | 0.07% | 0.07% | 0.07% | 0.07% | 0.07% | 0.07% | 0.07% | 0.07% | 0.07% |
| 40 | 0.08% | 0.08% | 0.08% | 0.08% | 0.08% | 0.08% | 0.08% | 0.08% | 0.08% | 0.08% |
| 41 | 0.08% | 0.08% | 0.08% | 0.08% | 0.08% | 0.08% | 0.08% | 0.08% | 0.08% | 0.08% |
| 42 | 0.09% | 0.09% | 0.09% | 0.09% | 0.09% | 0.09% | 0.09% | 0.09% | 0.09% | 0.09% |
| 43 | 0.10% | 0.10% | 0.10% | 0.10% | 0.10% | 0.10% | 0.10% | 0.10% | 0.10% | 0.10% |
| 44 | 0.11% | 0.11% | 0.11% | 0.11% | 0.11% | 0.11% | 0.11% | 0.11% | 0.11% | 0.11% |
| 45 | 0.12% | 0.12% | 0.12% | 0.12% | 0.12% | 0.12% | 0.12% | 0.12% | 0.12% | 0.12% |
| 46 | 0.13% | 0.13% | 0.13% | 0.13% | 0.13% | 0.13% | 0.13% | 0.13% | 0.13% | 0.13% |
| 47 | 0.14% | 0.14% | 0.14% | 0.14% | 0.14% | 0.14% | 0.14% | 0.14% | 0.14% | 0.14% |
| 48 | 0.15% | 0.15% | 0.15% | 0.15% | 0.15% | 0.15% | 0.15% | 0.15% | 0.15% | 0.15% |
| 49 | 0.16% | 0.16% | 0.16% | 0.16% | 0.16% | 0.16% | 0.16% | 0.16% | 0.16% | 0.16% |
| 50 | 0.18% | 0.18% | 0.18% | 0.18% | 0.18% | 0.18% | 0.18% | 0.18% | 0.18% | 0.18% |
| 51 | 0.19% | 0.19% | 0.19% | 0.19% | 0.19% | 0.19% | 0.19% | 0.19% | 0.19% | 0.19% |
| 52 | 0.21% | 0.21% | 0.21% | 0.21% | 0.21% | 0.21% | 0.21% | 0.21% | 0.21% | 0.21% |
| 53 | 0.23% | 0.23% | 0.23% | 0.23% | 0.23% | 0.23% | 0.23% | 0.23% | 0.23% | 0.23% |
| 54 | 0.25% | 0.25% | 0.25% | 0.25% | 0.25% | 0.24% | 0.24% | 0.24% | 0.24% | 0.24% |
| 55 | 0.27% | 0.27% | 0.27% | 0.27% | 0.26% | 0.26% | 0.26% | 0.26% | 0.26% | 0.26% |
| 56 | 0.29% | 0.29% | 0.29% | 0.28% | 0.28% | 0.28% | 0.28% | 0.28% | 0.28% | 0.28% |
| 57 | 0.31% | 0.31% | 0.31% | 0.31% | 0.31% | 0.30% | 0.30% | 0.30% | 0.30% | 0.30% |
| 58 | 0.33% | 0.33% | 0.33% | 0.33% | 0.33% | 0.33% | 0.33% | 0.32% | 0.32% | 0.32% |
| 59 | 0.36% | 0.36% | 0.36% | 0.35% | 0.35% | 0.35% | 0.35% | 0.35% | 0.35% | 0.35% |
| 60 | 0.39% | 0.39% | 0.38% | 0.38% | 0.38% | 0.38% | 0.38% | 0.38% | 0.37% | 0.37% |
| 61 | 0.42% | 0.41% | 0.41% | 0.41% | 0.41% | 0.41% | 0.40% | 0.40% | 0.40% | 0.40% |
| 62 | 0.45% | 0.45% | 0.44% | 0.44% | 0.44% | 0.44% | 0.43% | 0.43% | 0.43% | 0.43% |
| 63 | 0.49% | 0.48% | 0.48% | 0.48% | 0.47% | 0.47% | 0.47% | 0.47% | 0.46% | 0.46% |
| 64 | 0.53% | 0.52% | 0.52% | 0.51% | 0.51% | 0.51% | 0.50% | 0.50% | 0.50% | 0.50% |
| 65 | 0.58% | 0.57% | 0.56% | 0.56% | 0.56% | 0.55% | 0.55% | 0.55% | 0.54% | 0.54% |
| 66 | 0.63% | 0.63% | 0.62% | 0.62% | 0.61% | 0.61% | 0.60% | 0.60% | 0.60% | 0.59% |
| 67 | 0.70% | 0.69% | 0.69% | 0.68% | 0.68% | 0.67% | 0.67% | 0.66% | 0.66% | 0.66% |
| 68 | 0.78% | 0.77% | 0.76% | 0.75% | 0.75% | 0.74% | 0.74% | 0.73% | 0.73% | 0.73% |
| 69 | 0.86% | 0.85% | 0.84% | 0.84% | 0.83% | 0.82% | 0.82% | 0.81% | 0.81% | 0.81% |

Data for people aged 0 to 19 years is included as this was used in the dynamic model to estimate population projections over time.

Appendix 9: Formulae used to estimate mortality rates for people with and without obesity.

The following formulae was used to calculate the mortality rates for those with obesity and those who were non-obese (normal/overweight):

1. MortObese = MortNon-obese x HR
2. MortTot = MortObese x Prevobesity + Mort Non-obese x (1-Prevobesity)

MortTot = MortNon-obese x HR x Prevobesity + MortNon-obese x (1-Prevobesity)

MortNon-obese = MortTot / [HR x Prevobesity + (1-Prevobesity)]

Where:

MortTot is the mortality rate for the total population;

MortObese is the mortality rate for the sub-population with obesity;

MortNon-obese is the mortality rate for the non-obese sub-population;

Prevobesity is the prevalence of obesity; and

HR is the hazard ratio for mortality associated with obesity relative to non-obesity

Please note, the prevalence of obesity was specific to each year in the model and was calculated using population numbers in the normal weight, overweight and obese weight categories only (in line with the mortality hazard ratio data).

Appendix 10: Estimated equivalent full-time workers using the Australian Bureau of Statistics labour force statistics for a) males and b) females.

1. Males

| **Age group (years)** | **Employed** | **Mean number of hours worked** | **Unemployed** | **Not in the labour force*** | **Total number of equivalent full time workers (EFT)**** | **Proportion of equivalent full time workers (EFT) in the total population†** |
| --- | --- | --- | --- | --- | --- | --- |
| 15-19 | 254,118 | 20 | 70,449 | 366,502 | 127,059 | 18% |
| 20-24 | 510,658 | 32 | 76,674 | 156,340 | 408,526 | 55% |
| 25-29 | 622,212 | 39 | 49,215 | 91,939 | 606,657 | 79% |
| 30-34 | 671,460 | 41 | 37,143 | 71,897 | 688,247 | 88% |
| 35-39 | 623,792 | 42 | 31,475 | 67,158 | 654,982 | 91% |
| 40-44 | 622,889 | 42 | 31,317 | 73,668 | 654,033 | 90% |
| 45-49 | 609,023 | 42 | 30,929 | 82,867 | 639,474 | 88% |
| 50-54 | 571,887 | 42 | 30,512 | 97,601 | 600,481 | 86% |
| 55-59 | 500,078 | 40 | 29,642 | 133,130 | 500,078 | 75% |
| 60-64 | 349,759 | 37 | 25,473 | 215,270 | 323,527 | 55% |
| 65-69 | 169,675 | 33 | 6,103 | 365,555 | 139,982 | 26% |
| 70-74 | 64,911 | 29 | 1,962 | 332,195 | 47,060 | 12% |
| 75 and over | 37,305 | 27 | 1,042 | 584,762 | 25,181 | 4% |
| Total 20 to 69 |  |  | 348,483 | 1,355,425 | 5,215,987 | 75% |

1. Females

| **Age group (years)** | **Employed** | **Mean number of hours worked** | **Unemployed** | **Not in the labour force*** | **Total number of equivalent full time workers (EFT)**** | **Proportion of equivalent full time workers (EFT) in the total population†** |
| --- | --- | --- | --- | --- | --- | --- |
| 15-19 | 280,834 | 15 | 63,765 | 315,126 | 105,313 | 16% |
| 20-24 | 486,356 | 28 | 58,019 | 178,629 | 340,449 | 47% |
| 25-29 | 571,385 | 33 | 42,119 | 170,356 | 471,393 | 60% |
| 30-34 | 581,229 | 31 | 36,467 | 192,466 | 450,452 | 56% |
| 35-39 | 535,603 | 30 | 32,973 | 175,355 | 401,702 | 54% |
| 40-44 | 570,496 | 32 | 33,604 | 157,591 | 456,397 | 60% |
| 45-49 | 581,761 | 33 | 30,998 | 151,183 | 479,953 | 63% |
| 50-54 | 539,942 | 33 | 27,059 | 163,962 | 445,452 | 61% |
| 55-59 | 459,061 | 32 | 22,148 | 220,653 | 367,249 | 52% |
| 60-64 | 292,699 | 29 | 14,425 | 319,460 | 212,207 | 34% |
| 65-69 | 120,351 | 25 | 2499 | 444,922 | 75,219 | 13% |
| 70-74 | 38,292 | 22 | 811 | 383,383 | 21,061 | 5% |
| 75 and over | 18,051 | 21 | 616 | 785,627 | 9477 | 1% |
| Total 20 to 69 |  |  | 300,311 | 2,174,577 | 3,700,473 | 51% |

*The Australian Bureau of Statistics (ABS) defines “not in the labour force” as persons aged 15 years and over who are neither employed nor unemployed. Examples of those not in the labour force includes persons who are retired or voluntarily inactive; performing home duties or caring for children; attending an educational institution; experiencing a long-term health condition or disability; experiencing a short-term illness or injury; looking after an ill or disabled person; on a travel, holiday or leisure activity; working in an unpaid voluntary job; in institutions (hospitals, jails, sanatoriums, etc.); permanently unable to work; and members of contemplative religious orders.

**Total number of equivalent full-time (EFT) workers =

[mean number of working hours for each age category/40 (average full time working hours per week)] x total number of employed individuals for each age category

†Proportion of equivalent full-time (EFT) workers in the total population = total number of EFT workers /total number in the labour force (employed, unemployed and not in the labour force).

EFT assumes average full-time working hours of 40 hours per week.

Appendix 11: GDP per hour worked (projections derived from the Australian Bureau of Statistics trend data from 1975 to 2018).

|  | **2021** | **2022** | **2023** | **2024** | **2025** | **2026** | **2027** | **2028** | **2029** | **2030** |
| --- | --- | --- | --- | --- | --- | --- | --- | --- | --- | --- |
| GDP per hour worked | $103.9 | $105.1 | $106.3 | $107.5 | $108.7 | $109.9 | $111.1 | $112.3 | $113.5 | $114.7 |

Costs are reported in Australian dollars (AU$).

Appendix 12: The discounted years of life lived, PALYs and value of PALYs for the Australian working age from 2021 to 2030 for males and females assuming a) current trajectory of incident obesity, b) a 2% reduction in incident obesity and c) a 5% reduction in incident obesity.

1. Current trajectory of incident obesity

| **Year** | **Males** |  |  | **Females** | | |
| --- | --- | --- | --- | --- | --- | --- |
|  | **Years lived** | **PALYs** | **Value of PALYs** | **Years lived** | **PALYs** | **Value of PALYs** |
| 2021 | 7,732,337 | 5,697,428 | $1,136,987,651,671 | 7,851,620 | 3,939,487 | $786,170,219,413 |
| 2022 | 7,448,249 | 5,487,186 | $1,107,666,893,924 | 7,563,606 | 3,790,381 | $765,142,558,338 |
| 2023 | 7,171,001 | 5,281,870 | $1,078,383,695,480 | 7,282,451 | 3,645,405 | $744,271,559,472 |
| 2024 | 6,903,816 | 5,083,918 | $1,049,675,334,163 | 7,009,210 | 3,505,194 | $723,716,488,889 |
| 2025 | 6,645,745 | 4,893,716 | $1,021,673,221,072 | 6,744,021 | 3,370,240 | $703,613,288,026 |
| 2026 | 6,395,333 | 4,709,534 | $994,065,869,296 | 6,487,296 | 3,240,326 | $683,952,487,249 |
| 2027 | 6,154,265 | 4,531,008 | $966,817,129,936 | 6,239,861 | 3,115,476 | $664,773,785,805 |
| 2028 | 5,921,826 | 4,358,740 | $940,095,907,300 | 6,000,846 | 2,995,765 | $646,128,581,916 |
| 2029 | 5,696,386 | 4,192,799 | $913,960,498,028 | 5,769,218 | 2,880,464 | $627,893,386,956 |
| 2030 | 5,478,123 | 4,033,676 | $888,562,930,208 | 5,545,043 | 2,769,960 | $610,183,784,239 |
| Total | 65,547,081 | 48,269,875 | $10,097,889,131,078 | 66,493,172 | 33,252,698 | $6,955,846,140,302 |

1. A 2% reduction in incident obesity

| **Year** | **Males** |  |  | **Females** | | |
| --- | --- | --- | --- | --- | --- | --- |
|  | **Years lived** | **PALYs** | **Value of PALYs** | **Years lived** | **PALYs** | **Value of PALYs** |
| 2021 | 7,732,337 | 5,697,432 | $1,136,988,382,082 | 7,851,620 | 3,939,489 | $786,170,589,705 |
| 2022 | 7,448,250 | 5,487,197 | $1,107,669,083,396 | 7,563,607 | 3,790,386 | $765,143,653,919 |
| 2023 | 7,171,004 | 5,281,888 | $1,078,387,374,552 | 7,282,453 | 3,645,414 | $744,273,371,613 |
| 2024 | 6,903,824 | 5,083,943 | $1,049,680,501,723 | 7,009,214 | 3,505,206 | $723,718,999,990 |
| 2025 | 6,645,758 | 4,893,748 | $1,021,679,851,201 | 6,744,027 | 3,370,255 | $703,616,474,065 |
| 2026 | 6,395,351 | 4,709,572 | $994,073,906,889 | 6,487,305 | 3,240,344 | $683,956,314,510 |
| 2027 | 6,154,290 | 4,531,052 | $966,826,498,040 | 6,239,873 | 3,115,497 | $664,778,213,220 |
| 2028 | 5,921,856 | 4,358,789 | $940,106,520,274 | 6,000,861 | 2,995,788 | $646,133,566,501 |
| 2029 | 5,696,422 | 4,192,853 | $913,972,264,012 | 5,769,236 | 2,880,490 | $627,898,884,028 |
| 2030 | 5,478,165 | 4,033,735 | $888,575,757,027 | 5,545,064 | 2,769,987 | $610,189,750,610 |
| Total | 65,547,258 | 48,270,208 | $10,097,960,139,195 | 66,493,259 | 33,252,856 | $6,955,879,818,162 |

1. A 5% reduction in incident obesity

| **Year** | **Males** |  |  | **Females** | | |
| --- | --- | --- | --- | --- | --- | --- |
|  | **Years lived** | **PALYs** | **Value of PALYs** | **Years lived** | **PALYs** | **Value of PALYs** |
| 2021 | 7,732,337 | 5,697,437 | $1,136,989,477,698 | 7,851,620 | 3,939,492 | $786,171,145,142 |
| 2022 | 7,448,252 | 5,487,213 | $1,107,672,369,095 | 7,563,607 | 3,790,394 | $765,145,297,996 |
| 2023 | 7,171,010 | 5,281,915 | $1,078,392,898,911 | 7,282,456 | 3,645,428 | $744,276,092,519 |
| 2024 | 6,903,836 | 5,083,981 | $1,049,688,265,512 | 7,009,220 | 3,505,224 | $723,722,772,413 |
| 2025 | 6,645,778 | 4,893,796 | $1,021,689,817,632 | 6,744,036 | 3,370,278 | $703,621,262,875 |
| 2026 | 6,395,379 | 4,709,629 | $994,085,994,980 | 6,487,318 | 3,240,371 | $683,962,069,837 |
| 2027 | 6,154,326 | 4,531,118 | $966,840,593,636 | 6,239,891 | 3,115,528 | $664,784,873,975 |
| 2028 | 5,921,902 | 4,358,863 | $940,122,495,847 | 6,000,884 | 2,995,823 | $646,141,068,566 |
| 2029 | 5,696,476 | 4,192,934 | $913,989,982,390 | 5,769,263 | 2,880,528 | $627,907,160,602 |
| 2030 | 5,478,227 | 4,033,822 | $888,595,080,305 | 5,545,095 | 2,770,028 | $610,198,737,030 |
| Total | 65,547,524 | 48,270,709 | $10,098,066,976,006 | 66,493,390 | 33,253,094 | $6,955,930,480,956 |
